# The efficacy of ultrasound for plantar fasciitis, a systematic review and meta-analysis

**DOI:** 10.1080/07853890.2025.2543056

**Published:** 2025-08-10

**Authors:** Xuan Li, Jiao Yang, Shuhan Li, Liuxin Yang, Dianhuai Meng

**Affiliations:** Rehabilitation Center, the First Affiliated Hospital with Nanjing Medical University, Nanjing, Jiangsu, China

**Keywords:** Systematic review/meta-analysis, plantar fasciitis, ultrasound, pain measurement, rehabilitation

## Abstract

**Background:**

Ultrasound (US) has been widely used in the treatment of plantar fasciitis (PF); however, its therapeutic effect remains unclear.

**Objective:**

This meta-analysis aimed to investigate the effects of ultrasound (US) on pain intensity and foot function in patients with PF.

**Methods:**

PubMed, Web of Science, Cochrane Library, and other databases were systematically searched from inception to July 2024. Meta-analysis was performed using RevMan 5.3.

**Results:**

Thirteen trials were performed. There was no difference in pain relief between US alone and no treatment, as was US plus conventional physical exercises (CPE) compared with CPE alone. In the comparison between the US plus CPE group and the other interventions plus CPE group, there was a statistically significant difference in pain intensity (MD = 0.78, 95%CI= 0.12 to 1.44, *p* = 0.02), except in the case where extracorporeal shock wave therapy (ESWT) acted as an “other intervention” (MD = 0.75, 95%CI= −0.28 to 1.78, *p* = 0.15). The foot function measured in the Foot Function Index (FFI) showed a statistically significant difference between the two groups (MD =9.20, 95%CI = 0.77 to 17.63, *p* = 0.03), while the American Orthopaedic Foot showed no statistically significant difference.

**Conclusion:**

Whether applied alone or in combination with CPE, US cannot reduce pain intensity in patients with PF. However, US in combination with CPE may improve foot function.

## KEY MESSAGES

- The included studies reported the effects of US on pain intensity and foot function in patients with PF.
- This meta-analysis showed that US, either alone or in combination with CPE, could not reduce pain intensity in patients with PF.
- The role of US in the improvement of PF function should be paid more attention.

## Introduction

Plantar fasciitis (PF) is one of the most common foot conditions [1], and 11–15% of adults experience plantar pain throughout their life. Histology shows that plantar fasciitis may have both inflammatory and degenerative characteristics [2–4]. This can be attributed to overuse, excessive loading, and poor working habits [5–7]. PF is more likely to occur in patients with structural foot deformities than in those without. Patients usually experience increased pain in the morning or after a rest period. The pain decreased somewhat after walking but may recur after prolonged, sustained, or more vigorous activity [8]. Pain in PF is usually at the origin of the plantar fascia in the medial calcaneal tuberosity [2]. In more severely affected patients, passive dorsiflexion of the toes can aggravate pain.

Early treatment of PF usually involves non-steroidal anti-inflammatory drugs (NSAIDs), stretching of the gastrocnemius and plantar fascia, and use of orthoses (heel pads, heel cups, arch supports, or night splints). For foot pain that lasts for 6 months or longer, corticosteroid injection (CSI), botulinum toxin injection, dry needling, radiofrequency thermal lesioning (RTL), extracorporeal shock wave therapy (ESWT), and ultrasound (US) are typically used [9,10]. Numerous studies and evidence have shown that these physiotherapeutic interventions effectively relieve pain and improve foot function [11–13]. Surgery is also a treatment option, but none has shown good results [14]. When comparing the efficacy of these physiotherapeutic interventions on PF, the conclusions vary, which may be due to differences in dosage, intervention duration, and follow-up periods, reflecting the complexity of PF treatment.

Among these treatments, CSI, ESWT, and RTL are the three most preferred modalities used by clinical orthopedic surgeons, which are typically used in sequence. Erden et al. [15] studied the effects of CSI, ESWT and RTL on pain relief in PF, finding that all three significantly reduced pain scores, with CSI and RTL demonstrating better therapeutic effects. However, Ozan et al.’s [16] experiment found that EWST and RTL were equally effective in relieving pain. Yapici et al. [17] also observed that the efficacy of the three in terms of pain intensity was similar in patients.

Ultrasound (US) is a popular evidence-based intervention for a variety of clinical problems. The frequencies used in therapy are typically between 1.0 and 3.0 MHz. US has been widely used in tissue repair and wound healing over the past few years [18]. It can promote cell proliferation and angiogenesis, inhibit inflammatory cell infiltration, and accelerate healing. For US, ligaments, tendons, fascia, joint capsules, and scar tissue are the best absorptive tissues [19]. Therefore, US is often used in the traditional treatment of PF [20].

A recent systematic review [21] supported the use of US to reduce pain and improve foot function in patients with PF. However, this review did not include all possible associated studies; its data retrieval deadline was October 2022, and only five randomized control trials (RCTs) were finally enrolled. Meanwhile, the review did not conduct a meta-analysis of the data, and its conclusion was based only on the author’s comprehensive analysis.

The purpose of our review was to further clarify the role of US in pain relief and foot function in patients with PF with meta-analysis in the following groups: (a) US vs. no treatment, (b) US plus conventional physical exercise (CPE) vs. CPE alone, and (c) US plus CPE vs. other interventions plus CPE.

## Methods

This systematic review was conducted according to the Preferred Reporting Items for Systematic Reviews and Meta-Analyses (PRISMA) guideline [22]. This review was registered in PROSPERO (registered ID CRD42024505892).

### Study search and selection strategy

Three electronic databases (PubMed, Web of Science, and Cochrane Library) and other databases were comprehensively searched for RCTs and prospective trials from inception to July 2024 by two authors independently, without any language restrictions. Taking PubMed as an example, the following search terms were used for study retrieval: ((“Ultrasonic Therapy”[Mesh]) OR (((((((((Therapies, Ultrasonic[Title/Abstract]) OR (Ultrasonic Therapies[Title/Abstract])) OR (Therapeutic Ultrasound[Title/Abstract])) OR (Ultrasound, Therapeutic[Title/Abstract])) OR (Therapy, Ultrasonic[Title/Abstract])) OR (Ultrasound Therapy[Title/Abstract])) OR (Therapies, Ultrasound[Title/Abstract])) OR (Therapy, Ultrasound[Title/Abstract])) OR (Ultrasound Therapies[Title/Abstract]))) AND ((“Fasciitis, Plantar”[Mesh]) OR ((((((((((((Plantar Fasciitis[Title/Abstract]) OR (Policeman’s Heel[Title/Abstract])) OR (Heel, Policeman’s[Title/Abstract])) OR (Heels, Policeman’s[Title/Abstract])) OR (Policeman Heel[Title/Abstract])) OR (Policeman’s Heels[Title/Abstract])) OR (Policemans Heel[Title/Abstract])) OR (Heel Spur Syndrome[Title/Abstract])) OR (Chronic Plantar Fasciitis[Title/Abstract])) OR (Fasciitis, Chronic Plantar[Title/Abstract])) OR (Plantar Fasciitis, Chronic[Title/Abstract])) OR (Fasciitis, Plantar, Chronic[Title/Abstract]))).

Studies were included in our study if they met the following criteria: (a) study design included prospective trials and RCTs; (b) participants were diagnosed with PF; (c) one of the treatment arms must include US; (d) CPE must include daily stretching of the plantar fascia and calf muscles; and (e) there were no restrictions on sex, age, race, and severity of disease. Studies were excluded if they: (a) contained case-control studies, case series, and case reports; (b) enrolled less than 10 patients; and (c) could not provide data or did not include any outcomes we used in our study.

To ensure that only relevant and high-quality studies were included in our analysis, two authors independently evaluated the retrieved articles. Articles that met the inclusion criteria were selected through discussion. Disagreements were resolved by consensus with a third reviewer.

### Outcome measures and data extraction

The main outcome indicators of US for PF are pain intensity and foot function. Pain was measured using the visual analog scale (VAS) and numeric pain-rating scale (NPRS). Foot function was evaluated using the American Orthopaedic Foot and Ankle Society (AOFAS) scale score and Foot Function Index (FFI). A predefined Excel spreadsheet was used for data collection. The extracted information included the first author, year of publication, country, and study design (treatment, sample size). Any discrepancies in the data extraction were resolved by a third investigator.

### Quality and risk of bias assessments

The Cochrane Handbook for Reviews of Interventions (Revman Version 5.3) was used to assess the risk of bias. Two independent authors subjectively reviewed all articles and assigned a value of “high,” “low” or “unclear” based on the following items: random sequence generation, allocation concealment, blinding to participants, researchers and outcome evaluators, incomplete data, selective outcome reporting, and other sources of bias. Disagreements were resolved through discussion to reach a consensus. If consensus could not be reached, a third investigator was consulted.

### Statistical analysis

Statistical analysis was performed using the Review Manager version 5.3. The extracted data were entered into the Review Manager by two independent authors. The results are reported as forest plots with 95% confidence intervals (CIs). For continuous outcomes, the mean difference (MD) was calculated between the two groups. We used the *I^2^* index to examine heterogeneity across trials for each outcome. Studies with an *I^2^* statistic of 0–25% were considered to have low heterogeneity, those with an *I^2^* statistic of 25–75% were considered to have moderate heterogeneity, and those with an *I^2^* statistic > 75% were considered to have high heterogeneity. A fixed-effect model was used for meta-analysis if *I^2^* < 25% or *p* > 0.10. Otherwise, a random-effect model was used (*I^2^* > 25% or *p* < 0.10). Statistical significance was indicated by a *p*-value < 0.05.

## Results

### Description of literature screening and quality assessment

**Figure 1.**
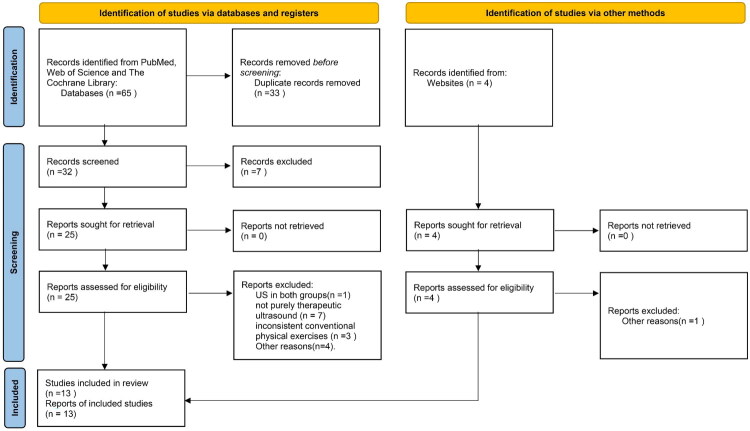
PRISMA flow diagram.

**Figure 2.**
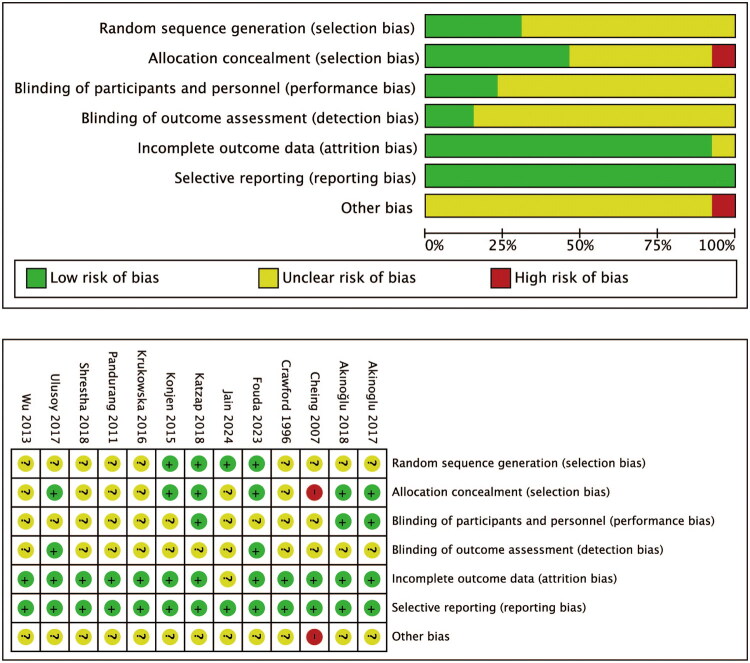
Risk of bias graph (upper) and summary (lower).

A total of 69 articles were identified. In total, 40 duplications were removed after scrutinizing the titles and abstracts. Then, 29 full-text articles were assessed for their eligibility. As shown in Figure 1, US in both groups (*n* = 1), not purely therapeutic ultrasound (*n* = 7), inconsistent conventional physical exercises (*n* = 3), and other reasons (*n* = 5) were excluded. Finally, 13 studies, involving 594 participants, were included in the meta-analysis. The characteristics of the included studies are shown in Table 1. All eligible studies were published in 1996 and mainly conducted in Asia. Of the 13 included studies, two [32,34] compared the improvement of PF with US alone versus no treatment. Five [23,28–31] studies compared the therapeutic effects of US combined with CPE and CPE alone in the treatment of PF. Nine [24–30,33,35] studies compared the efficacy of US plus CPE with other interventions plus CPE in the treatment of PF, two [24,25] of which involved three groups of treatment methods. The assessment of the risk of bias is shown in Figure 2.

**Table 1.** Characteristics of the included studies.

Study ID	Year	Country	Sample size	Male/female	Interventions	Treatment 1				Treatment 2				Treatment 3				Outcomes
						t1	parameter	Cases	Age, years	t2	parameter	Cases	Age, years	t3	parameter	Cases	Age, years	
Katzap et al. 2018 [23]	2018	Israel	54	18/36	US+CPE VS sham US+CPE	US+CPE	continuous, 1 MHZ, 1.8 W/cm2, 8 minutes	25	50.93 ± 12.87	sham US+CPE	pulsed, 3 MHZ, 0.1 W/cm2, duty cycle of 1:4, 8 minutes	25	52.58 ± 12.36					NPRS
Fouda et al. 2023 [24]	2023	Egypt	69	25/44	US+ CPE VS ESWT+CPE VS US+ESWT+CPE	US+ CPE	continuous, 1 MHz, 1.5 W/cm2, 5 minutes	23	41.63 ± 4.65	ESWT+CPE	8 Hz, 2.5 bar, 2,000 shoots.	23	40.96 ± 4.18	US+ESWT+CPE	US+SW	23	41.08 ± 4.45	FFI
Ulusoy et al. 2017 [25]	2017	Turkey	60	11/49	US+CPE VS ESWT+CPE VS LLLT+CPE	US+CPE	continuous, 1 MHz, 2.0 W/cm2, 5 minutes	17	50.95 ± 9.62	ESWT+CPE	10Hz, 2.5bar, 2000 shoots	20	54.45 ± 6.90	LLLT+CPE	8 J/cm2; 830 nm; 200 seconds	17	53.40 ± 14.71	VAS AOFAS
Krukowska et al. 2016 [26]	2016	Poland	47	NA	US+CPE VS ESWT+CPE	US+CPE	pulsed; 1 MHz; 1.5 W/cm2, 80 % fill factor, 4 minutes	20	51.10 ± 8.60	ESWT+CPE	10 Hz, 2.5 bar, 2000 shoots	27	51.40 ± 7.80					VAS
Konjen et al. 2015 [27]	2015	Thailand	30	6/24	US+CPE VS ESWT+CPE	US+CPE	continuous, 3MHz, 0.5-1 watt/cm2, 10 minutes.	15	45.00 ± 1.13	ESWT+CPE	10Hz, 2 bar, 2000 shoots	15	45.60 ± 1.07					VAS
Akınoğlu 2018 [28]	2018	Turkey	54	0/54	US+CPE VS ESWT+CPE VS CPE	US+CPE	plused, 3.0-MHz, 1 W/cm², 20% intermittently, 8 minutes	18	50.11 ± 9.29	ESWT+CPE	3.0 Hz, 0.2 mj/mm2, 1500 shoots; 8 Hz, 0.3 mj/mm², 300 shoots; each session 2000 pulses	18	50.00 ± 6.54	CPE	NA	18	45.22 ± 7.64	VAS
Akinoglu et al. 2017 [29]	2017	Turkey	54	0/54	US+CPE VS ESWT+CPE VS CPE	US+CPE	plused, 3.0-MHz, 1 W/cm², 20% intermittently, 8 minutes	18	50.11 ± 9.29	ESWT+CPE	3.0 Hz, 0.2 mj/mm2, 1500 shoots; 8 Hz, 0.3 mj/mm², 300 shoots; each session 2000 pulses	18	50.00 ± 6.54	CPE	NA	18	45.22 ± 7.65	FFI AOFAS
Wu et al. 2013 [30]	2013	China	30	9/21	US+CPE VS PH+CPE VS CPE	US+CPE	plused, 1 MHz, 1.0∼1.8W/cm2, 50％ intermittently, 10 minutes	10	45.80 ± 6.10	PH+CPE	plused, 1 MHz, 1.0∼1.8W/cm2, 50％ intermittently, 10 minutes + Futalin	10	48.40 ± 8.00	CPE	NA	10	46.7 ± 6.5	VAS FFI
Pandurang 2011 [31]	2011	India	30	15/15	US+CPE VS sham US+CPE	US+CPE	plused, 1 MHz, 0.5 W/cm2, a pulse ratio of 1:4, 8 minutes	15	41.80 ± 5.70	sham US+CPE	NA	15	41.93 ± 6.23					FFI
Cheing et al. 2007 [32]	2007	China	37	11/26	US VS ESWT VS no treatment	US	continuous, 1MHz, 1W/cm2, 5 minutes	15	48.80 ± 7.50	ESWT	4Hz, 0.23 to 0.37mJ/mm2, 1000 shoots	12	46.80 ± 10.30	no treatment	NA	10	43.30 ± 4.40	VAS
Shrestha 2018 [33]	2018	Nepal	60	23/37	US+CPE VS local injection steroid++CPE	US+CPE	continuous, 1MHz, 7 minutes	30	NA	local injection steroid + CPE	2 ml Methyl Prednisolone Acetate mixed with 0.5 ml of 1% Lignocain	30	NA					VAS FFI
Crawford 1996 [34]	1996	United Kingdom	19	NA	US VS sham US	US	plused, 3 MHz, 0.5 W/cm2, pulsed 1:4, 8minutes	13 heels	NA	sham US	NA	13 heels	NA					VAS
Jain 2024 [35]	2024	India	50	5/45	US+CPE VS dry needling therapy + CPE	US+ CPE	pulsed, 3 MHz, 8 minutes	25	NA	dry needling therapy + CPE	NA	25	NA					NPRS

Abbreviations: ultrasound: US, conventional physical exercises: CPE, extracorporeal shock wave therapy: ESWT, phonophoresis: PH, low-level laser therapy: LLLT.

### Ultrasound versus no treatment

**Figure 3.**
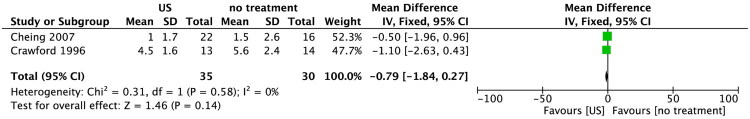
Forest plot of pain intensity in the US group compared with no treatment group. Abbreviations: US: ultrasound; CPE: conventional physical exercises; IV: inverse variance.

Two studies compared US with no treatment; both used the VAS to assess pain relief after treatment, as shown in Figure 3. There was no statistically significant difference in the MD analyses showing a lower level of pain intensity in the US group (MD = −0.79, 95%CI = −1.84 to 0.27, *p* = 0.14). A low level of heterogeneity was observed among the studies (*I^2^* = 0%, *p* = 0.58).

### Ultrasound plus conventional physical exercises versus conventional physical exercises alone

Five studies compared the efficacy of US plus CPE with that of CPE alone for the treatment of PF. Pain reduction after training was assessed by Akınoğlu and Köse [28], Wu et al. [30], and Katzap et al. [23],where Akınoğlu and Kose, Wu et al. used the VAS to assess pain intensity, and Katzap et al. used the NPRS. Foot function was evaluated by Akınoğlu et al. [29], Wu et al. [30], and Pandurang et al. [31] using FFI, while Akınoğlu et al. [29] used AOFAS to evaluate hind foot ability.

**Figure 4.**
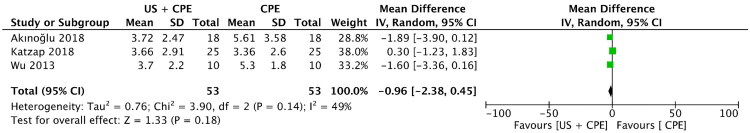
Forest plot of pain intensity in the US plus CPE group compared with CPE group. Abbreviations: US: ultrasound; CPE: conventional physical exercises; IV: inverse variance.

1. Pain intensity: In Figure 4, there was no statistically significant difference seen in the MD analyses, showing a higher pain reduction in the US plus CPE group (MD = −0.96, 95%CI = −2.38 to 0.45, *p* = 0.18). Moderate heterogeneity was found among the studies (*I^2^* = 49%, *p* = 0.14).
2. FFI: In the treatment of PF, Pandurang [31] used FFI to evaluate foot function, and both groups showed significant improvement (*p* < 0.05); however, the US plus CPE group showed statistically significant improvement compared to the CPE group (*p* < 0.05). Wu et al. [30] used the FFI disability subscale to determine foot function. The FFI decreased in all groups; however, after 1 month of treatment, the improvement in the US plus CPE group was significantly higher than that in the CPE group (*p* < 0.05). The FFI was used to show the patient’s pain level, disability, and activity limitations by Akınoğlu et al. [29]. After treatment, both groups showed a reduction in FFI pain, disability, and activity limitation subtitles (*p* < 0.05), but these were most marked in the US plus CPE group compared with the CPE groups (*p* < 0.05).
3. AOFAS: Akınoğlu et al. [29] used AOFAS to evaluate foot function. The AOFAS score of the hind legs increased in all groups, but the end value was lower in the CPE group than in the US plus CPE group (*p* < 0.05).

### Ultrasound plus conventional physical exercises versus other interventions plus conventional physical exercises

Of the nine studies comparing US plus CPE with other interventions plus CPE in treating PF, six [25–28,30,33] studies used the VAS to assess the improved condition after treatment for pain intensity and one [35] used the NPRS, of which Ulusoy et al. [25] used two other interventions to compare with US. In these studies, four articles [25–28] using ESWT plus CPE as the control group. In terms of foot functional improvement, two studies [24,33] used FFI, and Fouda et al. [24] included two other treatments; two studies [25,29] used AOFAS, and Ulusoy et al. [25] included three control groups.

**Figure 5.**
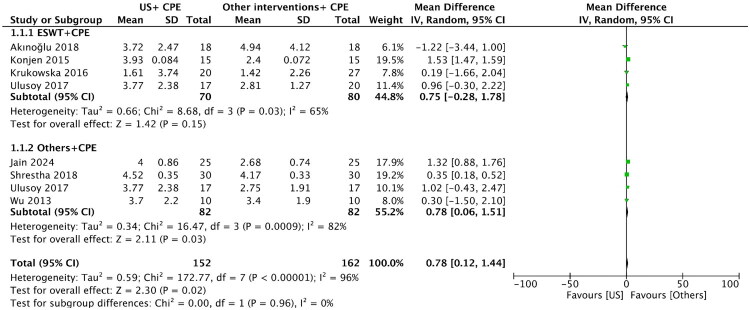
Forest plot of pain intensity in the US plus CPE group compared with other interventions plus CPE group. Abbreviations: US: ultrasound; CPE: conventional physical exercises; ESWT: extracorporeal shock wave therapy; IV: inverse variance.

**Figure 6.**
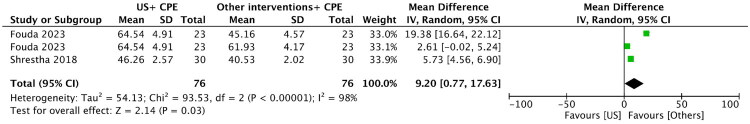
Forest plot of FFI in the US plus CPE group compared with other interventions plus CPE group. Abbreviations: US: ultrasound; CPE: conventional physical exercises; IV: inverse variance.

**Figure 7.**
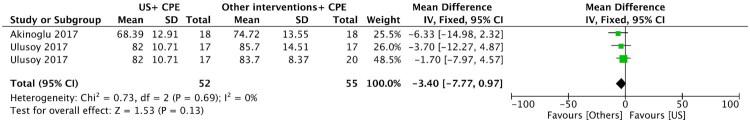
Forest plot of AOFAS in US plus CPE group compared with other interventions plus CPE group. Abbreviations: US: ultrasound; CPE: conventional physical exercises; IV: inverse variance.

1. Pain intensity: In Figure 5, there was a statistically significant difference seen in the MD analyses, showing a higher pain reduction in the other interventions plus CPE group (MD =0.78, 95%CI = 0.12 to 1.44, *p* = 0.02). A high level of heterogeneity was found among the studies (*I^2^* = 96%, *p* < 0.00001). Sensitivity analysis failed to determine any one or two trials that might have caused statistical heterogeneity. ESWT, as one of the other interventions, showed higher pain reduction than the US plus CPE group, but the difference was not statistically significant (MD =0.75, 95%CI = –0.28 to 1.78, *p* = 0.15).
2. FFI: In Figure 6, there was a statistically significant difference seen in the MD analyses, showing a lower FFI score in the other interventions plus CPE group compared to the US plus CPE group (MD =9.20, 95%CI =0.77 to 17.63, *p* = 0.03). A high level of heterogeneity was found among the studies (*I^2^* = 98%, *p* < 0.00001). Sensitivity analysis failed to determine any one or two trials that might have caused statistical heterogeneity.
3. AOFAS: In Figure 7, there was no statistically significant difference seen in the MD analyses, showing a higher AOFAS scale score for the other interventions plus CPE group (MD = −3.40, 95%CI = −7.77 to 0.97, *p* = 0.13). A low level of heterogeneity was observed among the studies (*I^2^* = 0%, *p* = 0.69).

## Discussion

### Effect of US on pain intensity

VAS scores between the US and no-treatment groups were not statistically significant (Figure 3), although VAS scores were lower in the US group. In the comparison between the US plus CPE and CPE groups, the US plus CPE group was not superior to the CPE group in terms of analgesia (Figure 4). PF guidelines in 2023 recommended not using US to enhance the benefits of CPE in treating PF because of Petrofsky’s research [2]. Petrofsky et al. [36] found that, in treating plantar foot pain, the VAS score in the heat group decreased significantly after a 4-hour treatment (pain from 53.91-mm SD ± 21.32 to 30.13-mm SD ± 26.81, *p*<.001). This treatment lasted for 4 h and was not applicable to the US.

US has two effects: thermal and non-thermal [37]. Thermal effects can relieve pain and improve the blood circulation. However, in treating PF, the heat absorbed by the tissue cannot cause the thermal effect of US in a short time. It also includes the non-thermal effects of cavitation and micromassage. Non-thermal effects lead to increased cell permeability and affect cell growth processes, thereby improving tissue healing [38]. Pulsed ultrasound (PUS) is the most dominant application of non-thermal effects. In recent years, many studies have focused on the potential effects of low-intensity pulsed ultrasound (LIPUS), which has a great influence on fracture repair, neuromodulation, soft tissue repair, and anti-inflammatory effects [39]. LIPUS has been found to relieve pain in knee osteoarthritis and periodontitis by reducing the expression of pro-inflammatory cytokines [38,40]. Among the 13 RCTs, Krukowska et al. [26],Wu et al. [30], Crawford and Snaith [34], Akınoğlu and Köse [28], and Astha Jain and Muthukumaran [35] used PUS to treat PF and found that the VAS score decreased significantly after treatment. Therefore, LIPUS may have a therapeutic effect on PF and is worthy of clinical attention.

In the comparison between the US plus CPE group and other interventions plus CPE group, there was a statistically significant difference in analgesia (Figure 5). In the analysis of other interventions plus CPE, we focused on these treatments: ESWT, dry needling therapy, local steroid injection, phonophoresis, and laser, of which ESWT had 4 groups. Among them, the analgesic effect of dry needling therapy, local steroid injection and laser plus CPE were better than that of US plus CPE, and the analgesic effect of phonophoresis plus CPE was equivalent to that of US plus CPE. In addition, when comparing ESWT plus CPE with US plus CPE, there was no statistically significant difference between the two groups. However, a network meta-analysis conducted by Li et al. [10] found that ESWT could induce significant pain reduction compared with placebo at 0 to 6 weeks (MD = 3.67, 95% CI = 0.31 to 6.9), and US might have good efficacy in a short-term period. This result was consistent with that reported by Li et al. [41]. Therefore, more attention should be devoted to other therapies to find a better intervention for the treatment of PF.

### Effect of US on foot function

Forest plot analysis showed that other interventions plus CPE improved foot function in patients with PF when evaluated using FFI (Figure 6). The effectiveness of US in improving foot function in plantar fasciitis (PF) may be due to its ability to accelerate tissue repair, increase the flexibility of collagen fibers, and reduce pain and muscle spasms [37]. However, the AOFAS score showed no statistically significant difference between the two groups (Figure 7). The FFI [42] is often used to measure the impact of foot pathology on function in terms of pain, disability, and activity restriction. It is a self-administered index consisting of 23 items on three subscales. The American Orthopaedic Foot Society (AOFAS) ankle-hindfoot score is one of the most commonly used tools to measure treatment outcomes in patients with complex ankle or hindfoot injuries. It combines both clinician and patient report sections [43]. The AOFAS and FFI both show similar criteria validity for similar diseases, but the objective components of AOFAS are more difficult to implement [44]. While Kostuj et al. [45] used the Oxford Foot Model to determine which of the two scores better correlates with objective gait dysfunction, they found that, compared with FFI, AOFAS showed a good agreement with objective gait parameters and is therefore more suitable for assessing disability and functional limitations in patients with foot and ankle disease. Further studies should be conducted to develop a questionnaire that can better represent plantar function.

In recent years, ESWT has gained greater attention [2]. However, Li et al. [46] and Al-Siyabi et al. [47] compared US with ESWT and found that ESWT showed a superior effect when compared with US in terms of functional impairment but failed to reach statistical significance. No physiotherapeutic interventions were found to be the most effective in improving plantar function in patients with PF. Thus, further studies should be conducted to identify the best physiotherapeutic intervention for improving foot function in patients with PF.

There were only three studies on the US plus CPE group versus the CPE alone group. Three studies used different evaluation methods for FFI, and only one group used AOFAS to evaluate hind foot function; therefore, meta-analysis could not be performed. However, when analyzed separately, Akınoğlu et al. [29], Wu et al. [30], and Pandurang [31] concluded that US plus CPE was beneficial for the functional improvement of PF. Thus, US plus CPE may be effective. However, further research is required to determine this pattern.

### Limitations

Similar to other meta-analyses, our study had some limitations. First, a total of 13 studies were included, and the number of included studies was relatively small, among which high-quality trials were relatively scarce. Second, differences in pain duration, US parameters (pattern, frequency, intensity, pulse ratio, and time), and outcome measures lead to high heterogeneity and different clinical outcomes. In summary, high-quality RCTs must be designed to confirm the therapeutic effects of US.

## Conclusions

This meta-analysis indicated that US could not reduce pain in PF, either alone or in combination with CPE. US combined with CPE may be used to improve foot function in patients with PF. Therefore, more attention should be paid to its efficacy. Considering the small sample size, further clinical trials are recommended to evaluate the present conclusions.

## Supplementary Material

PRISMA checklist.docx

## Data Availability

The authors confirm that the data supporting the findings of this study are available in the article and its supplementary materials, and further inquiries can be directed to the corresponding author.
